# Weather-Dependent Risk for Legionnaires’ Disease, United States

**DOI:** 10.3201/eid2311.170137

**Published:** 2017-11

**Authors:** Jacob E. Simmering, Linnea A. Polgreen, Douglas B. Hornick, Daniel K. Sewell, Philip M. Polgreen

**Affiliations:** The University of Iowa, Iowa City, Iowa, USA

**Keywords:** Legionnaires’ disease, pneumonia, temperature, weather, humidity, seasonality, antibiotic stewardship, bacteria, antimicrobial resistance, legionellosis, Legionella pneumophilia

## Abstract

Using the Nationwide Inpatient Sample and US weather data, we estimated the probability of community-acquired pneumonia (CAP) being diagnosed as Legionnaires’ disease (LD). LD risk increases when weather is warm and humid. With warm weather, we found a dose-response relationship between relative humidity and the odds for LD. When the mean temperature was 60°–80°F with high humidity (>80.0%), the odds for CAP being diagnosed with LD were 3.1 times higher than with lower levels of humidity (<50.0%). Thus, in some regions (e.g., the Southwest), LD is rarely the cause of hospitalizations. In other regions and seasons (e.g., the mid-Atlantic in summer), LD is much more common. Thus, suspicion for LD should increase when weather is warm and humid. However, when weather is cold, dry, or extremely hot, empirically treating all CAP patients for LD might contribute to excessive antimicrobial drug use at a population level.

Legionellosis is associated with a mild febrile illness, Pontiac fever, or Legionnaires’ disease (LD) (*1*), a cause of severe, atypical, community-acquired pneumonia (CAP) (*2*). *Legionella* spp. are aerobic, gram-negative bacilli, common in the environment, that were identified as pathogenic after an outbreak of illness among attendees of a 1976 American Legion convention (*1*,*3*). Although there are several species of *Legionella* and different serotypes, *L. pneumophilia* causes most LD cases (*4*,*5*). The case-fatality rate for LD among community-dwelling persons is as high as 10% (*5*). Delayed initiation of appropriate antimicrobial drug therapy further increases death rates (*6*,*7*), and the severity of LD drives the rationale for covering atypical organisms in the guidelines for empiric treatment of CAP. In developed countries, *Legionella* causes 1%–4% of CAP cases (*4*,*8*,*9*). Thus, because the rate of LD is low, many persons with CAP may be unnecessarily treated for LD. In fact, a recent noninferiority study, which included aggressive diagnostic testing, showed similar outcomes when treating and not treating for atypical organisms (*10*).

A striking epidemiologic feature of *Legionella*-associated CAP is its seasonality; more cases are reported during the summer (*1*). In contrast, hospital-associated cases do not exhibit seasonality (*1*). Seasonality has been described in the mid-Atlantic United States (*11*–*14*), England and Wales (*15*), and the Netherlands (*16*). Changes in use of cooling towers (*17*) or additional testing for pneumonia during the summer have been hypothesized as causes of this seasonality (*1*). However, strong evidence indicates that weather, particularly temperature and humidity, drive the summer spike in incidence (*11*,*12*,*15*,*16*). Although *Legionella* spp. are common in the environment, dry environments do not support them (*1*), and *Legionella* spp. are more sensitive than other pathogens to drying conditions (*18*). In contrast, warm and humid weather tends to support pathogen survival, growth, and the potential for aerosol exposures, increasing disease risk (*1*,*13*,*19*).

If the incidence of LD depends on local weather, the baseline rate of LD might be extremely low year-round in some locations and during specific seasons in other locations. Use of local weather data ultimately might provide information to help determine whether a specific CAP case is caused by *Legionella*. To establish the risk for LD across season, location, and weather conditions, we combined patient-level data on hospitalizations for pneumonia and LD from 26 US states with local weather data.

## Methods

### Data Source and Case Definition

**Figure 1:**
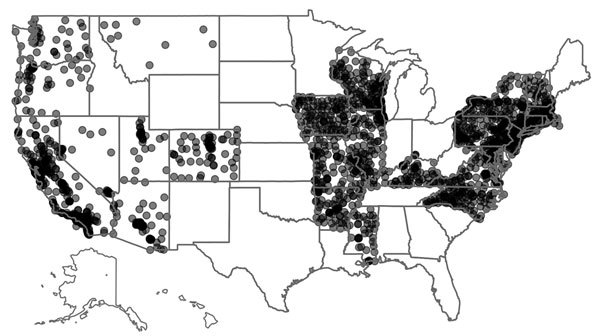
Locations of Healthcare Cost and Utilization Project (HCUP) hospitals used in the analysis of risk for Legionnaires’ disease, 26 US states, 1998–2011. Because many hospitals are near each other, each hospital was plotted as a faint point. When multiple points overlap, the area becomes darker because of the stacking of the points. Thus, there are faint spots in more rural areas and dark clusters in more urban areas.

We extracted individual-level inpatient-event data from the Agency for Healthcare Research and Quality’s Healthcare Cost and Utilization Project (HCUP) Nationwide Inpatient Sample (NIS) for 1998–2011. The University of Iowa Institutional Review Board deems such studies as non–human subjects research. The NIS, a stratified 20% sample of discharges from nonfederal US hospitals, contains data from 47 states; after excluding the 21 NIS states that do not report the American Hospital Association identifier (AHA ID), patient race, or admission month, we used data from 26 states: Arizona, Arkansas, California, Colorado, Connecticut, Illinois, Iowa, Kentucky, Maryland, Massachusetts, Mississippi, Missouri, Montana, Nevada, New Hampshire, New Jersey, New York, North Carolina, Oregon, Pennsylvania, Rhode Island, Utah, Vermont, Virginia, Washington, and Wisconsin. Next, we mapped the hospitals in these 26 states to the AHA-reported addresses using the AHA ID; we then converted the addresses to geographic coordinates by using the US Census Bureau Geocoder (https://www.census.gov/geo/maps-data/data/geocoder.html) and Google Maps’ Geocoding API (Google; Mountain View, CA, USA). We located 2,079 unique hospitals (Figure 1).

We identified LD cases as hospitalizations of persons with a primary diagnosis code of 482.84 (pneumonia due to Legionnaires’ disease) from the International Classification of Diseases, Ninth Revision, Clinical Modification (ICD-9-CM). The primary diagnosis in the NIS is the condition chiefly responsible for the hospital admission (*20*). The sensitivity and specificity of the 482.84 code for LD was previously evaluated in a New York, NY, hospital for 2003–2013 (*21*); the authors reported high sensitivity (83.5%) and specificity (99.9%), a positive predictive value of 88.0%, a negative predictive value of 99.8%, and agreement between the estimated cases observed in the NIS for 2012 and the Centers for Disease Control and Prevention (CDC) data (*21*).

As study controls, we used hospitalizations of persons with a primary diagnosis ICD-9-CM code of 481 and subcodes (pneumococcal pneumonia) and 482 and subcodes (other bacterial pneumonia) excluding 482.84 (pneumonia due to Legionnaires’ disease). We refer to the combination of the codes 481 and 482 as bacterial pneumonia. Because these codes were assigned to patients as their primary diagnosis code for admission, we assumed that this collection of codes identified cases of community-acquired bacterial pneumonia. Finally, using CDC surveillance results (*22*), we computed the correlation between the national-level estimated number of LD cases in our data and the number reported by CDC (*23*).

We excluded records for persons <18 years of age and records that omitted any of our variables of interest: age, sex, payer, race, admission month and year, and hospital location. We also required the hospital to have >1 weather station within 100 km (62 miles).

### Weather Definition

We obtained weather observations from the Integrated Surface Database (ISD) provided and maintained by the National Climatic Data Center of the National Oceanic and Atmospheric Administration. Because the NIS database provides only the month of admission, we aggregated the average temperature, relative humidity, and total rainfall by month for each weather station. We recorded each hospital’s monthly weather data as the mean of these values observed at nearby (within 100 km [62 miles]) weather stations. Using only the states with hospital location reported, we considered different definitions of “nearby.” Average temperatures computed using only the nearest station and stations within 10 or 25 miles were highly correlated with the average temperature using a 62-mile radius (r>0.99).

### Modeling

Using logistic regression, we modeled whether a hospitalization for bacterial pneumonia had a diagnosis of LD on the basis of patient age, patient sex, payer, patient race, admission month, admission year, hospital latitude, total monthly rainfall, mean relative humidity, mean temperature, and an interaction between temperature and relative humidity. The interaction is required because relative humidity depends on temperature. We used mean temperature because it captures the nighttime and daytime temperature effects more accurately than does mean high temperature. However, the average high temperature and the average temperature for a month are highly correlated (r = 0.988). We used relative humidity rather than absolute humidity for 2 reasons. First, relative humidity ranges from 0% to 100% for any temperature, whereas absolute humidity ranges from 0 g/m^3^ to some temperature-specific maximum, which introduces problems because often the lowest observed absolute humidity at high temperatures is impossible at lower temperatures. Second, the values of absolute humidity were extremely correlated with temperature (r = 0.85), whereas the correlation was much lower with relative humidity (r = −0.23). The correlation of temperature with relative humidity is negative because relative humidity is often much higher at cold temperatures. To make the results easier to interpret and because the expected responses are not linear, we converted humidity and temperature into bins: relative humidity <50.0%, 50.0%–55.0%, 55.1%–60.0%, 60.1%–65.0%, 65.1%–70.0%, 70.1%–75.0%, 75.1%–80.0%, and >80.0%; and mean temperature <60°F, 60.1–80°F, >80°F. Additionally, monthly rainfall was binned into dry (<18 mm, the lowest 25%), normal (18–85.85 mm, the middle 50%), and wet (>85.85 mm, the top 25%). Patient age was binned by decade, and we included hospital latitude and squared hospital latitude. The squared hospital latitude was included to enable the effect of latitude to be nonlinear.

To visualize the model and how LD risk varies with space and season, we computed the fitted values from this model using location and weather information and set the demographic variables to their individual modal values for cases of bacterial pneumonia observed in the NIS data. Because the weather data are nationally complete, unlike the NIS data, we can take a given demographic profile (e.g., white man, 68 years of age, on Medicare) and estimate the probability of an LD diagnosis for any location, given the weather data for each location and month.

## Results

The NIS data provided a total of 5,172 LD cases from 447,132 hospitalizations for bacterial pneumonia (Table 1). After applying the discharge weights to produce a national-level estimate and before applying any exclusion rules other than reporting month and year, the NIS series compares favorably to CDC’s reported monthly LD estimates (*23*), with a correlation of 0.74. After applying exclusion rules (age >18 years and provision of a complete set of predictor variables), we had data on 3,005 LD cases and 189,412 hospitalizations for pneumonia. The most common reasons for exclusion were lack of the AHA ID, admission month and year, and race, because some states elected not to report these variables.

**Table 1:** Sample sizes for Legionnaires’ disease cases and other pneumonia controls in a study of weather-dependent risk for Legionnaires’ disease, United States, 1998–2011*

Characteristics reported	No. (% of initial sample)	
Cases	Controls

Total	5,172 (100.0)	447,132 (100.0)
Age >18 y	5,158 (99.7)	418,086 (93.5)
AHA ID	4,039 (78.1)	288,427 (64.5)
Sex	4,039 (78.1)	288,427 (64.5)
Payer	4,034 (78.0)	287,847 (64.4)
Admission month and year	3,542 (68.5)	253,725 (56.7)
Race	3,006 (58.1)	189,630 (42.4)
Weather station within 100 km (62 mi) of hospital	3,005 (58.1)	189,412 (42.4)

*The analysis comprised data from 26 states: Arizona, Arkansas, California, Colorado, Connecticut, Illinois, Iowa, Kentucky, Maryland, Massachusetts, Mississippi, Missouri, Montana, Nevada, New Hampshire, New Jersey, New York, North Carolina, Oregon, Pennsylvania, Rhode Island, Utah, Vermont, Virginia, Washington, Wisconsin. AHA ID, American Hospital Association identifier.

We compared several demographic and severity measures between the portion of the data dropped because of missing values and the proportion retained (Table 2). Among the cases, the only substantial difference was in the percentage of patients not insured; for 10.8% of dropped records, no insurance was reported, compared with 7.4% of those used in the model. Many statistically significant differences existed between the controls retained and those lost; however, the large sample size (retained sample n = 189,412) meant many non–clinically relevant differences would be statistically significant.

**Table 2:** Demographic and severity characteristics among dropped and retained records in a study of weather-dependent risk for Legionnaires’ disease, United States, 1998–2011*

Characteristics	Cases				Controls		
	Dropped, n = 2,153	Retained, n = 3,005	p value		Dropped, n = 228,674	Retained, n = 189,412	p value
Mean age, y (± SD)	60.6 (15.7)	61.8 (15.6)	0.0078		68.2 (17.1)	68.8 (17.2)	<0.0001
Female, %	39.6	39.1	0.7138		48.1	48.2	0.4170
Privately insured, %	39.0	38.8	0.8814		19.6	17.0	<0.0001
Not insured, %	11.2	7.7	<0.0001		6.2	4.5	<0.0001
Mean no. diagnoses (± SD)	9.6 (4.1)	9.6 (4.3)	0.9213		8.2 (3.9)	9.4 (4.3)	<0.0001
Mean no. procedures (± SD)†	1.9 (2.5)	1.9 (2.8)	0.6027		1.0 (1.8)	1.4 (2.2)	<0.0001

*Many of the significant differences in the controls resulted from the large sample and might not be clinically significant. The analysis comprised data from 26 states: Arizona, Arkansas, California, Colorado, Connecticut, Illinois, Iowa, Kentucky, Maryland, Massachusetts, Mississippi, Missouri, Montana, Nevada, New Hampshire, New Jersey, New York, North Carolina, Oregon, Pennsylvania, Rhode Island, Utah, Vermont, Virginia, Washington, Wisconsin. †Any type of procedure recorded in the Agency for Healthcare Research and Quality’s Healthcare Cost and Utilization Project; this was a measure of severity, and this variable was not included in the model.

In general, hospitalized persons with LD were younger than those with other bacterial pneumonia and more likely to be male (Table 3). We found a large unadjusted difference in the mean monthly environmental temperatures between cases (58.5°F) and controls (52.6°F), and monthly environmental relative humidity was higher on average for cases (70.0%) than for controls (67.3%). Additionally, there was nearly 20 mm more rain for cases (80.4 mm) than for controls (61.7 mm).

**Table 3:** Key variables in the sample in a study of weather-dependent risk for Legionnaires’ disease, United States, 1998–2011*

Variable	Cases, n = 3,005	Controls, n = 189,412
Age, y (± SD)	61.80 (15.61)	68.83 (17.15)
Sex, %		
F	39.13	48.18
M	60.87	51.82
Race/ethnicity, %		
White	76.64	79.60
Black	14.81	9.52
Hispanic	4.66	6.06
Other	3.89	4.83
Payer, %		
Medicare	45.82	69.22
Medicaid	7.69	9.27
Private	38.80	16.97
Uninsured	4.89	2.63
Other	2.80	1.92
Mean latitude, °N (± SD)	40.02 (2.75)	38.86 (3.30)
Mean monthly temperature, °F (± SD)	58.49 (14.96)	52.61 (15.32)
Mean monthly relative humidity, % (± SD)	70.03 (9.22)	67.34 (10.45)
Mean monthly total rainfall, mm (± SD)	80.39 (69.15)	61.68 (144.67)

*The analysis comprised data from 26 states: Arizona, Arkansas, California, Colorado, Connecticut, Illinois, Iowa, Kentucky, Maryland, Massachusetts, Mississippi, Missouri, Montana, Nevada, New Hampshire, New Jersey, New York, North Carolina, Oregon, Pennsylvania, Rhode Island, Utah, Vermont, Virginia, Washington, Wisconsin.

In the regression analysis, the primary variables of interest—mean temperature, mean relative humidity, and their interaction—were all significant (likelihood ratio test against model with only main effects, χ^2^ test statistic = 350.42; p<0.0001) (Table 4). Although total rainfall was independently a risk factor for LD, the effects of temperature and humidity were still significant.

**Table 4:** Effects of humidity and temperature on Legionnaires’ disease risk, United States, 1998–2011*

Variable	Odds ratio	95% CI
Age, y		
18–30	1.00	Ref
31–40	2.60	2.30–2.95
41–50	2.23	2.87–3.62
51–60	3.22	2.88–3.61
61–70	2.29	2.04–2.57
71–80	1.74	1.54–1.96
81–90	1.37	1.21–1.55
>91	0.78	0.66–0.93
Race/ethnicity		
White	1.00	Ref
Black	1.37	1.31–1.44
Hispanic	1.05	0.96–1.13
Other	1.00	0.93–1.10
Payer		
Medicare	1.00	Ref
Medicaid	0.87	0.81–0.93
Private	2.43	2.32–2.53
Uninsured	1.86	1.71–2.03
Other	1.65	1.48–1.83
Sex		
M	1.00	Ref
F	0.75	0.73–0.78
Hospital admission mo		
Jan	1.00	Ref
Feb	0.85	0.77–0.94
Mar	0.74	0.67–0.82
Apr	0.91	0.82–1.00
May	1.10	0.99–1.23 1.35–1.71
Jun	1.52	
Jul	2.24	1.99–2.52
Aug	2.49	2.21–2.80
Sep	2.35	2.10–2.64
Oct	2.58	2.37–2.80
Nov	1.63	1.49–1.79
Dec	1.14	1.04–1.25
Year	1.06	1.06–1.07
Hospital latitude, °N		
Latitude	3.06	2.67–3.50
Latitude squared	0.99	0.99–0.99
Total monthly rainfall†		
Dry	1.00	Ref
Normal	1.36	1.29–1.44
Wet	1.47	1.38–1.57
Mean monthly temperature, °F		
<60	1.00	Ref
60–80	0.55	0.44–0.68
>80	1.57	1.24–1.98
Mean RH, %		
0–50.0	1.00	Ref
50.1–55.0	0.85	0.68–1.06
55.1–60.0	0.58	0.48–0.70
60.1–65.0	0.66	0.56–0.78
65.1–70.0	0.79	0.67–0.93
70.1–75.0	0.80	0.68–0.94
75.1–80.0	0.76	0.64–0.90
80.1–100.0	0.65	0.53–0.78
Interactions		
Temperature 60°–80°F		
RH <50%	1.0	Ref
RH 50.1%–55.0%	1.65	1.19–2.28
RH 55.1%–60.0%	1.54	1.13–2.09
RH 60.1%–65.0%	2.15	1.68–2.75
RH 65.1%–70.0%	2.27	1.83–2.83
RH 70.1%–75.0%	2.93	2.36–3.64
RH 75.1%–80.0%	3.27	2.62–4.08
RH 80.1%–100.0%	4.79	3.71–6.17
Temperatures >80°F		
RH <50%	1.0	Ref
RH 50.1%–55.0%	0.00	–
RH 55.1%–60.0%	0.00	–
RH 60.1%–65.0%	1.23	0.77–1.95
RH 65.1%–70.0%	0.62	0.42–0.91
RH 70.1%–75.0%	0.38	0.24–0.60
RH 75.1%–80.0%	0.91	0.54–1.54
RH 80.1%–100.0%	0.00	–

*Based on a multivariable logit model with a diagnosis of Legionnaires’ disease as the outcome variable. The analysis comprised data from 26 states: Arizona, Arkansas, California, Colorado, Connecticut, Illinois, Iowa, Kentucky, Maryland, Massachusetts, Mississippi, Missouri, Montana, Nevada, New Hampshire, New Jersey, New York, North Carolina, Oregon, Pennsylvania, Rhode Island, Utah, Vermont, Virginia, Washington, Wisconsin. Dash indicates 95% CIs not defined because there were no data for cases or controls in that particular strata. †Dry, <18 mm; normal, 18–85.85 mm; wet, >85.85 mm.

The combination of the temperature and humidity main effects and interactions can make understanding the combined effect difficult. For this reason, we separately reported the estimated composite odds ratios for each combination (Table 5). Additionally, because odds ratios can be difficult to interpret, we provide the expected probabilities for a 61–70-year-old white man on Medicare admitted to a hospital at 42°N in April 2011 at the 3 different rainfall levels (Table 6). The relationship between temperature and relative humidity exhibits the Goldilocks principle: when it is too hot (>80°F) or too cold (<60°F), the odds of LD do not vary with humidity, but when the temperature is “just right” (60°–80°F), the odds have a dose-response pattern with humidity. The largest effect of this relationship between temperature and humidity was evident for warm and very humid months across all 3 rainfall levels.

**Table 5:** Odds ratios for Legionnaires’ disease based on the interaction between average monthly temperature and average monthly relative humidity, United States, 1998–2011*

Relative humidity, %	Average monthly temperature, °F		
	<60	60–80	>80
0–50	1.00	0.55	1.57
50.1–55.0	0.85	0.77	0.00
55.1–60.0	0.58	0.49	0.00
60.1–65.0	0.66	0.78	1.28
65.1–70.0	0.79	0.98	0.77
70.1–75.0	0.80	1.29	0.48
75.1–80.0	0.76	1.37	1.09
80.1–100.0	0.65	1.70	0.00

*Estimated from the multivariable logit model shown in Table 4.The analysis comprised data from 26 states: Arizona, Arkansas, California, Colorado, Connecticut, Illinois, Iowa, Kentucky, Maryland, Massachusetts, Mississippi, Missouri, Montana, Nevada, New Hampshire, New Jersey, New York, North Carolina, Oregon, Pennsylvania, Rhode Island, Utah, Vermont, Virginia, Washington, Wisconsin.

**Table 6:** Estimated probability of Legionnaires’ disease given bacterial pneumonia in a 61–70-year-old white man on Medicare located at 42°N, admitted to a hospital in April 2011*

	Probability of Legionnaires’ disease by rainfall level										
Relative	<60°F				60°F–80°F				>80°F		
humidity, %	Dry	Normal	Wet		Dry	Normal	Wet		Dry	Normal	Wet
0.0–50.0	1.46	1.98	2.13		0.81	1.10	1.18		2.28	3.08	3.31
50.1–55.0	1.24	1.68	1.81		1.13	1.53	1.64		0	0	0
55.1–60.0	0.86	1.16	1.25		0.72	0.98	1.06		0	0	0
60.1–65.0	0.97	1.32	1.42		1.15	1.56	1.68		1.86	2.52	2.71
65.1–70.0	1.16	1.57	1.69		1.44	1.95	2.10		1.13	1.53	1.64
70.1–75.0	1.17	1.59	1.72		1.88	2.54	2.74		0.71	0.96	1.03
75.1–80.0	1.12	1.52	1.63		1.99	2.69	2.90		1.59	2.16	2.32
80.1–100.0	0.95	1.29	1.39		2.46	3.32	3.57		0	0	0

*Because of the presence of month, latitude, and year in the model, these predicted probabilities are valid only on a line along 42°N in April 2011. The predicted values for other months (e.g., July or December) will differ, as will the predicted values for locations further north or south than 42°N. Dry, <18 mm; normal, 18–85.85 mm; wet, >85.85 mm.

**Figure 2:**
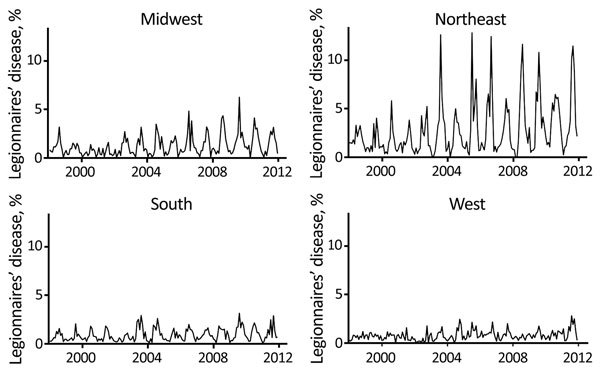
Time series of Legionnaires’ disease as a percentage of bacterial pneumonia discharges in Healthcare Cost and Utilization Project hospitals, 26 US states, 1998–2011. The Legionnaires’ disease series is highly seasonal in the Northeast, Midwest, and South. There are few cases and a lack of apparent seasonality in the West. The changes in the Legionnaires’ disease series after 2002–2003 may result from increased vigilance, testing, and reporting of atypical pneumonias (*24*).

We determined the monthly percentage of bacterial pneumonia discharges for which an LD diagnosis had been given within HCUP by US Census region (Figure 2). Percentages were relatively high in the Northeast and somewhat lower in the Midwest and South. The frequencies in the West were the lowest of all 4 regions and appeared not to be seasonal. The changes around 2002–2003 in all of the series are present in other data sources (*23*). The exact cause is unknown but is thought to be related to increased vigilance, testing, and reporting of atypical pneumonia after the outbreak of severe acute respiratory syndrome (*24*).

**Figure 3:**
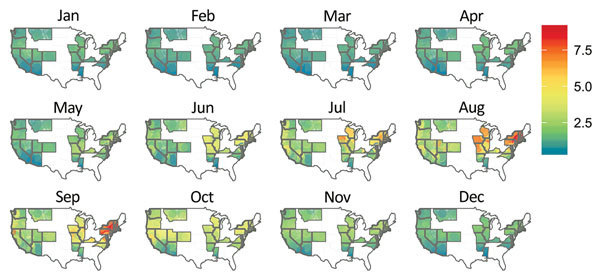
Predicted probability of an inpatient hospitalization for bacterial pneumonia being coded as Legionnaires’ disease by location and month in 2011 for 26 US states. The predicted risk is for a 61–70-year-old white man on Medicare (the most common Legionnaires’ disease patient in the data) by location in the United States for each month in 2011. These fixed covariates and actual monthly temperature, relative humidity, and latitude for each weather station in the Integrated Surface Database dataset were used to produce estimated probabilities using the model described in Table 4.

**Figure 4:**
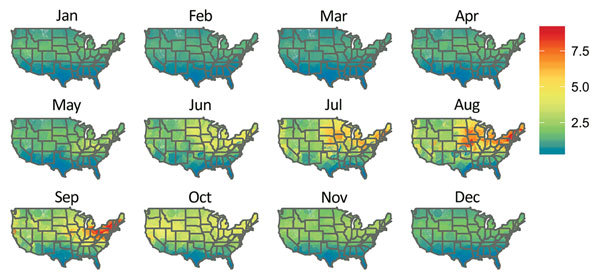
Predicted probability of an inpatient hospitalization for bacterial pneumonia being coded as Legionnaires’ disease, all US states, 2011. The predicted risk is for a 61–70-year-old white man on Medicare (the most common patient in the pooled case–control sample) by location for each month in 2011. These fixed covariates and actual monthly temperature, relative humidity, and latitude for each weather station in the Integrated Surface Database dataset were used to produce estimated probabilities using the model described in Table 4.

We also determined the probability of a case of bacterial pneumonia being diagnosed as LD in 2011 using the local weather data. We restricted this prediction to the states used to estimate the model (Figure 3). We set the nonweather, nonlocation covariates to their modal values for patients hospitalized with bacterial pneumonia (white 61–70-year-old man on Medicare) and used the weather station latitude and monthly average temperature, humidity, and rainfall. The estimated probabilities of LD (Figure 3) are the fitted values from the model described by Table 4 and these covariate values. Since the ISD is national in scope, we extrapolated from the model estimated using HCUP data to the entire United States, including non-HCUP regions (Figure 4). The risk for LD varied considerably by location (low risk along the Gulf Coast and relatively low risk in the West) and calendar month (high–risk areas such as the Mid-Atlantic region are only actually at high risk during June–September and are at low risk during December–April) (Figure 4).

## Discussion

Our results suggest that the incidence of LD varies considerably by season and local weather patterns. Specifically, LD is more likely to occur in warm (60°–80°F) and very humid (>80.0%) months. For example, the odds of LD being diagnosed in a pneumonia patient during a month when the rainfall is <18 mm and the temperature is 60°–80°F was 3.1 (1.70/0.55) times higher when the relative humidity was >80.0% than when it was <50.0%. When rainfall amounts were greater, the risk also increased; however, regardless of rainfall, warm and humid weather was a major risk factor. Also, we found a dose-response relationship between relative humidity and the odds of an LD diagnosis during periods of warm weather. In contrast, hot, cool, or dry weather patterns produce no meaningful increase in LD.

Previous work has demonstrated seasonality and the effects of weather patterns on LD (*1*,*11*–*13*,*15*,*16*). However, much of this work was based on regional investigations where LD is common. Regional investigations are limited in their ability to more fully describe the relationship between weather patterns and LD incidence. For instance, in the Rocky Mountains or the US Southwest, community-associated LD is comparatively rare, and the rate for LD is much lower than what would be expected given patient factors. In contrast, the Mid-Atlantic has a higher-than-expected risk during certain months of the year.

Using the national scope of our data, we estimated the differences that weather has on LD risk. The predicted probabilities of LD varied from 0% to 1% of all cases of bacterial pneumonia in the Southwest to nearly 8% during warm, humid, and rainy summer months in the mid-Atlantic. Weather appears to drive these differences because our model had no geographic information other than latitude. This finding is consistent with prior regionally and seasonally limited studies (*11*–*13*,*15*,*16*). Areas previously studied (e.g., the mid-Atlantic) vary considerably in risk depending on recent weather. Our results are biologically plausible because *L. pneumophilia* thrives in warm, wet environments (*25*), which support not only the pathogen’s survival but also the existence of aerosolizations. In contrast, conditions are not as supportive for the pathogen in dry or excessively hot environments (*1*,*18*).

LD is difficult to diagnose on the basis of clinical manifestations alone (*26*). Furthermore, rapid diagnostic tests do not cover all strains (*27*), and some tests have relatively low sensitivity (*28*). More definitive culture results may take 3–5 days after therapeutic decisions are needed (*1*). Thus, incorporating local weather conditions into clinical decision-making ultimately might help increase or decrease clinical suspicion for LD, especially when combined with diagnostic testing. Current US CAP guidelines recommend empiric therapy routinely covering atypical pneumonias (*29*). Results of a recent noninferiority trial suggest that monotherapy with a β-lactam, aggressive diagnostic testing, and use of clinical judgment may safely avert the use of fluoroquinolones or dual therapy with a macrolide in patients with CAP (*10*). However, the same trial replicated elsewhere with a higher rate of LD might yield different results. Another study investigating the effect of a β-lactam alone versus a β-lactam with a macrolide found delays in clinical stability for persons treated with only 1 agent, but the authors failed to show that the β-lactam alone was not inferior (*30*). Our model suggests that warm, humid, and rainy summer months in the mid-Atlantic may exhibit predicted probabilities of LD of nearly 8%. Accordingly, abandoning initial empiric coverage for LD might yield a differential effect on outcomes depending on season, region, and weather, and treating all CAP cases for atypical pneumonia in areas and seasons when LD is relatively uncommon may result in the excessive use of antimicrobial agents.

The antimicrobial drugs most commonly used to treat LD include either a fluoroquinolone or a macrolide (with a β-lactam), and resistance has increased for both (*10*,*31*–*34*). Thus, treating for LD only when and where risk is higher, along with increased diagnostic testing and good clinical judgement, may help reduce antimicrobial drug use, providing a new antimicrobial drug stewardship target. The temporal, climatologic, and geographic variations in LD risk emphasize the potential importance of regionally relevant guidelines. Basing treatment guidelines on estimates in high- or low-risk areas will lead to overuse or underuse of LD treatment for CAP. However, future work with more detailed clinical information is required to determine when and where antimicrobial drug use may be initially restricted and in which patients, without significantly impairing the quality of care. Specifically, future work will need to determine under which conditions and in which patients initial monotherapy with a β-lactam will not be inferior to combination therapy. Furthermore, future work will need to consider and incorporate diagnostic testing for LD and the patient’s clinical status (e.g., intensive care unit admission).

Our work has several limitations. First, our study uses administrative data for inpatient hospitalizations. Some CAP and LD cases are treated on an outpatient basis. Furthermore, state participation in the NIS is voluntary, and some states omit key variables (e.g., AHA IDs). Although we found no meaningful differences between the parts of the sample we retained and parts we dropped, it is possible that participation/reporting varies nonrandomly. In addition, we do not have information about medications, test results, or microbiology data, so we cannot confirm an LD diagnosis or determine whether and what microbiological testing was performed. Thus, future work should incorporate alternative case-finding approaches, including more granular information about cases, specifically the CDC legionellosis database. Alternative data sources may also enable the generation of more granular geographic estimates and age-based estimates of disease risk. We also are unable to confirm without more clinical data the extent to which having a primary diagnosis of pneumonia correlates with CAP for the control patients in our analysis. Second, some of the geographic differences in the predicted probabilities may be due to differences in propensity to test for, and therefore diagnose, LD. Third, the NIS reports only the month of admission. Thus, we aggregated weather information by month. Future work needs to consider more granular (e.g., daily) data. Fourth, we consider the weather around a hospital, not the weather experienced by the patients admitted to the hospital. Fifth, meteorologic variables are interdependent: relative humidity depends on temperature because the maximum amount of water suspended in the air rises with the temperature, and our model may inadequately specify these relationships. Finally, our analysis did not contain possibly important geographic differences (e.g., use of monochloramine in municipal water).

The experience of warm and humid weather patterns common during summer resulting in substantial increases in LD might have driven the current view about the frequency of LD in the United States. Our results demonstrate the need to investigate the effects of incorporating recent weather patterns, particularly wet, warm, and humid weather, as an additional consideration in the clinical decision-making process for CAP. Our results suggest that the risk for LD is highly related to temperature and humidity regionally. We found locations where LD relatively rarely causes hospitalization for CAP, such as the Southwest and Rocky Mountains, but also the Mid-Atlantic region during the winter. Information about weather exposures for patients also should help inform the design and interpretation of CAP-treatment trials. Future work examining more granular environmental data may ultimately enable clinicians to safely limit initial empiric antimicrobial drug selection for CAP to monotherapy with a β-lactam in specific seasons and regions.
